# Generation and analysis of the improved human HAL9/10 antibody phage display libraries

**DOI:** 10.1186/s12896-015-0125-0

**Published:** 2015-02-19

**Authors:** Jonas Kügler, Sonja Wilke, Doris Meier, Florian Tomszak, André Frenzel, Thomas Schirrmann, Stefan Dübel, Henk Garritsen, Björn Hock, Lars Toleikis, Mark Schütte, Michael Hust

**Affiliations:** Technische Universität Braunschweig, Institut für Biochemie, Biotechnologie und Bioinformatik, Spielmannstr. 7, 38106 Braunschweig, Germany; mAb-factory GmbH, Gelsenkirchenstr. 5, 38108 Braunschweig, Germany; YUMAB GmbH, Rebenring 33, 38106 Braunschweig, Germany; Klinikum Braunschweig g GmbH, Institut für Klinische Transfusionsmedizin, Celler Str. 38, 38114 Braunschweig, Germany; Department Vaccinology, Helmholtz-Zentrum für Infektionsforschung, Inhoffenstraße 7, 38124 Braunschweig, Germany; Merck KGaA, Darmstadt, Germany

**Keywords:** scFv, Phage display, Antibody engineering, Library, Panning, Screening

## Abstract

**Background:**

Antibody phage display is a proven key technology that allows the generation of human antibodies for diagnostics and therapy. From naive antibody gene libraries - in theory - antibodies against any target can be selected. Here we describe the design, construction and characterization of an optimized antibody phage display library.

**Results:**

The naive antibody gene libraries HAL9 and HAL10, with a combined theoretical diversity of 1.5×10^10^ independent clones, were constructed from 98 healthy donors using improved phage display vectors. In detail, most common phagemids employed for antibody phage display are using a combined His/Myc tag for detection and purification. We show that changing the tag order to Myc/His improved the production of soluble antibodies, but did not affect antibody phage display. For several published antibody libraries, the selected number of kappa scFvs were lower compared to lambda scFvs, probably due to a lower kappa scFv or Fab expression rate. Deletion of a phenylalanine at the end of the CL linker sequence in our new phagemid design increased scFv production rate and frequency of selected kappa antibodies significantly. The HAL libraries and 834 antibodies selected against 121 targets were analyzed regarding the used germline V-genes, used V-gene combinations and CDR-H3/-L3 length and composition. The amino acid diversity and distribution in the CDR-H3 of the initial library was retrieved in the CDR-H3 of selected antibodies showing that all CDR-H3 amino acids occurring in the human antibody repertoire can be functionally used and is not biased by *E. coli* expression or phage selection. Further, the data underline the importance of CDR length variations.

**Conclusion:**

The highly diverse universal antibody gene libraries HAL9/10 were constructed using an optimized scFv phagemid vector design. Analysis of selected antibodies revealed that the complete amino acid diversity in the CDR-H3 was also found in selected scFvs showing the functionality of the naive CDR-H3 diversity.

## Background

Since the inception of antibody technology twenty years ago, phage display is a powerful tool to generate antibodies for proteome research [1-4], diagnostics [5-8] or for therapeutic purposes [9-11]. Therapeutic antibodies are currently one of the fastest developing class of biologicals in the pharmaceutical market [12]. The main indications for therapeutic antibodies are cancer and auto-immune diseases [13,14]. To date, 44 antibodies and antibody conjugates are EMA and/or FDA approved (status autumn 2014) (http://www.imgt.org/mAb-DB/index) and about 350 antibodies and antibody fusion proteins were under development in 2013 [15]. Two major strategies for generating fully human antibodies are: transgenic mice and antibody phage display. In transgenic mice, the chromosome segments encoding antibody gene fragments are replaced with the corresponding human chromosome segments encoding human immunoglobulins. These animals allow the generation of fully human antibodies by hybridoma technology [16-18]. An advantage of transgenic mice is the *in vivo* affinity maturation of antibodies, but on the other hand, all *in vivo* antibody generations are restricted by the natural immune system itself: The limitation in antigen processing and presentation and the tolerance against conserved epitopes [19]. Antibody phage display is an alternative or complementing technology to generate human antibody fragments from universal antibody gene libraries as lead candidates for therapeutic development [17,20-22]. Here, the selection is an *in vitro* process and is not limited by the restrictions of the immune system and selection conditions can be adjusted and controlled, thus allowing to select for properties not achievable by *in vivo* immune systems [23]. To isolate human antibodies by phage display, two types of antibody gene libraries are used: immune libraries and universal or “single-pot” libraries [24,25]. Immune libraries from patients are suited to select specific antibodies against a disease or pathogen, e.g. cancer [26,27], human immunodeficiency virus [28]or herpes simplex virus [29]. “Single-pot” libraries allow the selection of antibodies - in theory - against any target. The human naive antibody gene libraries HAL4/7/8 are “single-pot” libraries. Antibodies against a panel of different antigens were selected from these HAL libraries and applied for different purposes, e.g. [8,30-35]. Antibody fragments from these libraries can directly be cloned into a selection of compatible expression vectors to produce e.g. *in vivo* biotinylated antibodies [31], scFv-Fc [36] or full IgG (Frenzel et al. unpublished). The scFv-Fc format (Yumab) is an alternative, functionally identical to IgG in most assays. Due to its quicker and easier production, it provides a robust format for screening of large numbers of antibody candidates, and can be converted to full IgG afterwards.

In this work, the scFv phagemid vector design was optimized and the “single-pot” antibody gene libraries HAL9/10 were constructed and analyzed, demonstrating significant improvements over previous designs.

## Methods

### Construction of phage display vectors

The phage display vector pHAL30 was constructed by cloning a DNA fragment encoding His-/C-Myc tag flanked by NotI and BamHI, which was generated with two PCR primers (MHMycHIS-NotI_f:5′ CGCGTGCGGCCGCAGGTTCTGAACAAAAGCTGATCTC 3′; MHMycHisBamHi_r: 5′ CGCGTGGATCCCTAATGATGATGGTGATGATGGG 3′) into pHAL14. ScFv coding sequences from pHAL14 were cloned in pHAL30 using the restriction sites NcoI and NotI. The C-terminal phenylalanine of the V_К_ was deleted from scFv coding sequences by PCR using the primer set MkpelB_f (5′ GCCTACGGCAGCCGCTGG 3′) and MhkappaCLscFv-NotI_r2 (5′ ACCGCCTCCGCGGCCGCGACAGATGGTGCAGCCACAGT 3′). The PCR products were cloned into the vector pHAL14 [31] and pHAL30 between the vector encoded signal peptide and the c-Myc/ His-tag or His/ c-Myc-tag sequences, respectively, using the restriction sites NcoI and NotI.

### Production of soluble antibodies in microtitre plates

96-well MTPs with polypropylene (PP) wells (U96 PP 0,5 mL, Greiner, Frickenhausen, Germany) containing 150 μL 2 × YT-GA [37]⁠ were inoculated with the bacteria bearing scFv expressing phagemids. MTPs were incubated overnight at 37°C at 800 rpm in a MTP shaker (Thermoshaker PST-60HL-4, Lab4You, Berlin, Germany). A volume of 180 μL phosphate-buffered 2 × YT-GA in a PP-MTP well was inoculated with 10 μL of the overnight culture and grown at 37°C and 800 rpm for 2 h. Bacteria were harvested by centrifugation for 10 min at 3,220 × g and 180 μL supernatants were removed. The pellets were resuspended in 180 μL 2 × YT supplemented with 100 μg/mL ampicillin, 100 mM sucrose and 50 μM isopropyl-beta D thiogalacto pyranoside (IPTG) and incubated at 30°C and 800 rpm overnight. Bacteria were pelleted by centrifugation for 10 min at 3,220 × g and 4°C. The scFv-containing supernatant was transferred to a new PP-MTP and stored at 4°C before analysis.

### Production of antibody phage

50 mL 2 × YT medium + 100 μg/mL ampicillin + 100 mM glucose were inoculated with an overnight culture to O.D._600_ = 0.01. Bacteria were grown to O.D._600_ = 0.4–0.5 at 37°C and 250 rpm. 2 mL bacteria (∼1 × 10^9^ bacteria) were infected with 2 × 10^10^ helper phage Hyperphage [38,39], incubated at 37°C for 30 min without shaking, followed by 30 min at 250 rpm. Infected cells were harvested by centrifugation for 10 min at 3,220 × *g* and the pellet was resuspended in 30 mL 2 × YT + 100 μg/mL ampicillin + 50 μg/mL kanamycin. Phage were produced at 30°C and 250 rpm for 16 h. Cells were pelleted for 10 min at 3,220 × *g*. Phage in supernatant were precipitated with 1/5 volume of 20% PEG/2.5 M NaCl solution for 1 h on ice and pelleted by centrifugation 1 h at 3,220 × *g* at 4°C. Precipitated phage were resuspended in 300 μL phage dilution buffer, and cell debris was pelleted by additional centrifugation for 5 min at 15,400 × *g* at 20°C. The supernatant containing the scFv phage was stored at 4°C. Phage titration (cfu) was done according to [40].

### Antigen ELISA

100 ng of antigen was coated to 96 well microtitre plates (MaxiSorp, Nunc) in PBS pH 7.4 or 50 mM NaHCO_3_ pH 9.6 overnight at 4°C. After coating, the wells were washed three times with PBST and blocked with 2%MPBST for 1.5 h at RT, followed by three washing steps with PBST. 10^8^ cfu scFv-phage and helperphage as control were loaded in the first well of each line of the plate. Each sample was diluted 1:2 in PBS along the plate ending with 100 μL proteinaceous solution per well. The loaded plate was incubated for 1.5 h at RT, followed by three PBST washing cycles. Bound scFv phage were detected with mouse anti-M13 HRP conjugate (Amersham Biosciences, Freiburg, Germany) (1:5,000 diluted in 2%MPBST). Visualization was performed using TMB (3,3′,5,5′-tetramethylbenzidine) as a substrate and the staining reaction was stopped by adding 100 μL 1 N sulphuric acid. Absorbance at 450 nm (620 nm reference) was measured by using a SUNRISE™ microtitre plate reader (Tecan, Crailsheim, Germany).

### SDS-PAGE

Antigens were analyzed by 12% SDS-PAGE using a Protean II Minigel system (BioRad Inc, München, Germany) according to [37]*.* Protein gels were stained with coomassie brilliant blue.

### Western blotting and immunostaining

Protein samples in SDS polyacrylamide gels were transferred to and immobilized on polyvinylidene difluoride (PVDF) membranes (Roth, Karlsruhe) by a semidry procedure (Biorad, München). The membrane was blocked with 2% (w/v) skimmed milk powder in PBST for 1 h at RT. ScFvs were detected by mouse anti c-myc mAb Myc1-9E10 (1:1,000 diluted in 2% MPBST) (Yumab, Braunschweig) for 1.5 h at RT and goat anti-mouse HRP mAb (A0168, Sigma, Taufkirchen) (1:3,200 diluted in 2%MPBST) for 1.5 h at RT. For visualization Super Signal® WestPico Chemiluminescence Substrate (Thermo Scientific, Bonn) was applied and protein bands were detected by enhanced chemiluminescence (ECL) on a luminometer (Biorad, München). For detection of antibody phage, the minor coat protein pIII was detected with 1:2,000 diluted mouse mAb anti-pIII (Mobitec, Göttingen) for 1.5 h at RT, followed by goat anti-mouse (Fc specific) mAb (Sigma, Taufkirchen) conjugated with AP (1:10,000 diluted). The development was performed by NBT/BCIP.

### Library construction

The study was performed in accordance with the Declaration of Helsinki. The study participants were selected from blood donors of the Institute for Clinical Transfusion medicine, Städtisches Klinikum Braunschweig gGmbH, Braunschweig, Germany. All voluntary donors were informed about the project and gave their informed consent. The use of blood samples for the amplification of antibody gene fragments to develop antibody phage display libraries was approved by the ethical commitee of the Technische Universität Braunschweig (Ethik-Kommission der Fakultät 2 der TU Braunschweig, approval number DM-2014-08).

Lymphocytes were isolated from 54 donors with various ethnical backgrounds using the Lymphoprep Kit (Progen, Heidelberg) according to the manufacturers instructions. Total RNA was isolated using Trizol (Invitrogen, Karlsruhe) and mRNA was isolated using the Oligotex mRNA Minikit (Qiagen, Hilden). Then cDNA was synthesized using Superscript III (Invitrogen), random hexamer oligonucleotide primers and 50–250 ng mRNA.

To amplify the antibody gene fragments (kappa, lambda, VH) 27 individual first PCRs of each cDNA sample were performed separately for each cDNA preparation in a volume of 50 μL using GoTaq (Promega, Mannheim) and 0.4 μM of each primer [40] for 30 cycles (1 min 95°C, 1 min 55°C, 2 min 72°C) followed by a 10 min final synthesis step. The PCR products were purified by agarose gel electrophoresis using the NucleoSpin Gel and PCR Clean-up Kit (Macherey-Nagel, Düren). VH, kappa or lambda PCR products were pooled separately. To add restriction sites for cloning, 100 ng of the purified and pooled VH, kappa or lambda PCR products of the first PCR for each of the 27 second PCR reactions were used in a volume of 100 μL using GoTaq and 0.2 μM of each primer for 20 cycles (1 min 95°C, 1 min 57°C, 2 min 72°C) followed by a 10 min final synthesis step at 72°C. PCR products were purified by agarose gel electrophoresis using the NucleoSpin Gel and PCR Clean-up Kit (Macherey-Nagel, Düren). VH, kappa or lambda PCR products were pooled separately. In total 1458 first PCR and 1458 second PCR reactions were performed.

**Table 1:** **Production of soluble scFv antibody fragments randomly chosen from the HAL8 antibody gene library**

**Production level**	**Clone**										
	**SWI6-2**	**SWI6-10**	**SWI6-14**	**SWI6-26**	**SWI6-30**	**SWI6-50**	**SWI6-51**	**SWI6-52**	**SWI6-55**	**SWI6-60**	**SWI6-46**
wt	0	++	++	+	++	+++	+++	++	+	+	+
dF	++	++	+++	+++	++	+++	+	++	++	+	++
**Production level**	**Clone**										
	**SWI6-48**	**SWI28-1**	**SWI28-2**	**SWI28-3**	**SWI6-4**	**SWI28-5**	**SWI28-6**	**SWI28-7**	**SWI28-8**	**SWI128-9**	**SWI128-10**
wt	+	++	++	+	+	+++	++	++	+	+	+
dF	++	+	0	+++	++	+++	++	++	++	+	+++
**Production level**	**Clone**										
	**SWI28-11**	**SWI28-12**	**SWI28-13**	**SWI28-14**	**SWI28-15**	**SWI28-16**	**SWI28-17**	**SWI28-18**	**SWI28-19**	**SWI28-20**	
wt	+	+	+++	+	0	0	+++	+	+	+++	
dF	+++	+	+++	++	++	+	+++	+	+	+++	

The scFvs were detected by Western blot analysis in 10 μL supernatant from *E.coli* XL-1 Blue MRF’ production in 96 well microtiter plates. The production yield was evaluated according to the band intensities. +++ best yield, ++ medium yield, + low yield, 0 no yield. Wt wild-type scFv coding sequence, dF deletion of the c-terminal phenylalanine from the scFv coding sequence.

For VL cloning 5 μg vector pHAL30 and 2 μg kappa or lambda PCR products were digested in a volume of 100 μL using 30 U *Mlu*I and *Not*I (NEB, Frankfurt) at 37°C for 3 h. This step was performed multiple times to produce enough material for up to 50 transformations. For quality control each digestion was performed separately with each enzyme in parallel. The digestions were inactivated at 80°C for 20 min, followed by adding 0.5 U calf intestine phosphatase (CIP) (MBI Fermentas, St. Leon-Rot) and incubation at 37°C for 30 min, this step was repeated once. Vector and PCR products were purified using the NucleoSpin Gel and PCR Clean-up Kit, removing short stuffer fragments between *Mlu*I and *Not*I in pHAL30. Each ligation was performed with 1 μg digested vector and 270 ng PCR product using 3 U T4 ligase (Promega, Mannheim) in a volume of 100 μL overnight at 16°C. Ligations were inactivated at 70°C for 10 min and precipitated with 10 μL 3 M sodium acetate pH 5.2 and 250 μL ethanol for 2 min at RT, followed by 5 min centrifugation at 16000 × g and 4°C. Pellet was washed two times with 500 μL 70% (v/v) ethanol. The dried pellet was resuspended in 35 μL dH_2_O and mixed on ice with 25 μL electrocompetent *E. coli* XL1-Blue MRF’. Electroporation was performed with 0.1 cm prechilled cuvettes using a MicroPulser electroporator (BioRad, München) at 1.7 kV. Immediately, 1 mL prewarmed 37°C SOC medium pH 7.0 (2% (w/v) tryptone, 0.5% (w/v) yeast extract, 0.05% (w/v) NaCl, 20 mM Mg solution, 20 mM glucose) was added and incubated for 1 h at 600 rpm and 37°C. Transformation was plated out on 25 cm square Petri dishes with 2xYT-GAT agar (1,6% (w/v) Tryptone, 1% (w/v) yeast extract, 0,5% (w/v) NaCl, 100 mM glucose, 100 μg/mL ampiciline, 20 μg/mL tetracycline, 1.5% (w/v) agar-agar) and incubated overnight at 37°C. In parallel 10^−6^ dilutions of each transformation reaction were plated out to determine the transformation rate. These plates were used also for quality control by colony PCR using the oligonucleotide primers MHLacZ-Pro_f and MHgIII_r to verify the insert size. The colonies were scraped from the 25 cm plates using 40 mL 2xYT medium (1,6% (w/v) Tryptone, 1% (w/v) yeast extract, 0,5% (w/v) NaCl) and 5 mL were directly used for midi plasmid preparation using the Nucleobond Plasmid Midi Kit (Macherey-Nagel).

For VH cloning material from 44 donors of the previous described HAL7/8 libraries [31] was used in addition to material of 54 new donors. In a first step 5 μg pHAL30-VL sublibraries and 2 μg VH PCR products were digested in a volume of 100 μL using 30 U *Nco*I-HF and 30 U *Hind*III-HF (NEB) at 37°C for 3 h. This step was performed several times to produce enough material for 150–200 transformations. The digestions, ligations and transformations were performed as described for VL cloning with following modifications. Ligation was performed with 250 ng VH PCR product. After transformation of XL1-Blue MRF’, overnight incubation on 25 cm 2 × YT-GAT plates and resuspension in 25 mL 2 × YT medium, glycerol stocks of each library were made using 800 μL bacteria solution (~10^10^ bacteria) and 200 μL glycerol (Roth, Karlsruhe) and stored at −80°C. For the final library one glycerol stock of each sublibrary was thawed, mixed together, aliquoted (1 mL) and stored at −80°C.

The antibody gene libraries HAL9/10 were packaged separately inoculating 400 mL 2 × YT-GA (2 × YT containing 100 mg/mL ampicillin and 100 mM glucose) with a library glycerol stock. The bacteria were grown to an optical density at 600 nm (OD600) of 0.4–0.5 at 37°C and 250 rpm. 25 mL bacterial culture (~1.25 × 10^10^ cells) were infected with 2.5 × 10^11^ Hyperphage particles [38], incubated at 37°C for 30 min without shaking followed by 30 min at 250 rpm. Infected cells were harvested by centrifugation for 10 min at 3220xg. The pellet was resuspended in 400 mL 2 × YT-AK (2 × YT containing 100 mg/mL ampicillin and 50 mg/mL kanamycin). Phage were produced at 30°C and 250 rpm over night. The bacteria were centrifuged for 20 min at 10,000 × g. Phage particles in the supernatant were precipitated with 1/5 volume of 20% (w/v) polyethylene glycol (PEG)/2.5 M NaCl solution for one hour on ice with gentle shaking and pelleted by centrifugation for one hour at 10,000 × g at 4°C. The precipitated phage were resuspended in 10 mL phage dilution buffer (10 mM TrisHCl pH 7.5, 20 mM NaCl, 2 mM EDTA), 1/5 volume of PEG solution was added and incubated on ice for 20 min, followed by centrifugation for 30 min at 10,000 × g and 4°C. The pellet was resupended in 1 mL phage dilution buffer. Residual bacteria and cell debris were removed by additional centrifugation for 5 min at 16000 × g at 20°C. Supernatants containing antibody phage were stored at 4°C. Phage titration was done as described before [41]. The scFv display rates of the packaged libraries were analyzed by 10% SDS-PAGE, Western blot and anti-pIII immunostain (mouse anti-pIII 1:2000, goat anti-mouse IgG AP conjugate 1:10000). Wildtype pIII has a calculated molecular mass of 42.5 kDa, but it runs at an apparent molecular mass of 65 kDa in SDS-PAGE [42]. Accordingly, the scFv::pIII fusion protein runs at about 95 kDa.

### Antibody selection (panning and screening)

Antibody selections were performed against proteins or peptides as described before [40]. The elution with trypsin - pHAL30 and the former vector pHAL14 contain a trypsin site between the tags and gIII - is more efficient as elution by pH shift (Schirrmann, unpublished results).

## Results

### Analysis of the new phage display vector pHAL30

**Figure 1:**
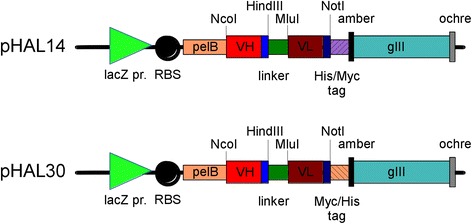
**Schematic drawing of pHAL14 and pHAL30.** Abbreviations: lacZ promoter: promoter of the bacterial lac operon; RBS: ribosome binding site; pelB: signal peptide sequence of bacterial pectate lyase, mediating secretion into the periplasmic space; VH: variable fragment of the heavy chain; blue box: first six amino acids of CH1, part of the linker; LC: light chain; dark blue box: first 9 (kappa)/16 (lambda) aminoacids of CL; ochre: ochre stop codon; amber: amber stop codon; His-tag: 6xhistidine tag; terminator: sequence terminating transcription; Myc-tag: EQKLISEEDLN tag. The elements of the inserts are not drawn to scale.

**Figure 2:**
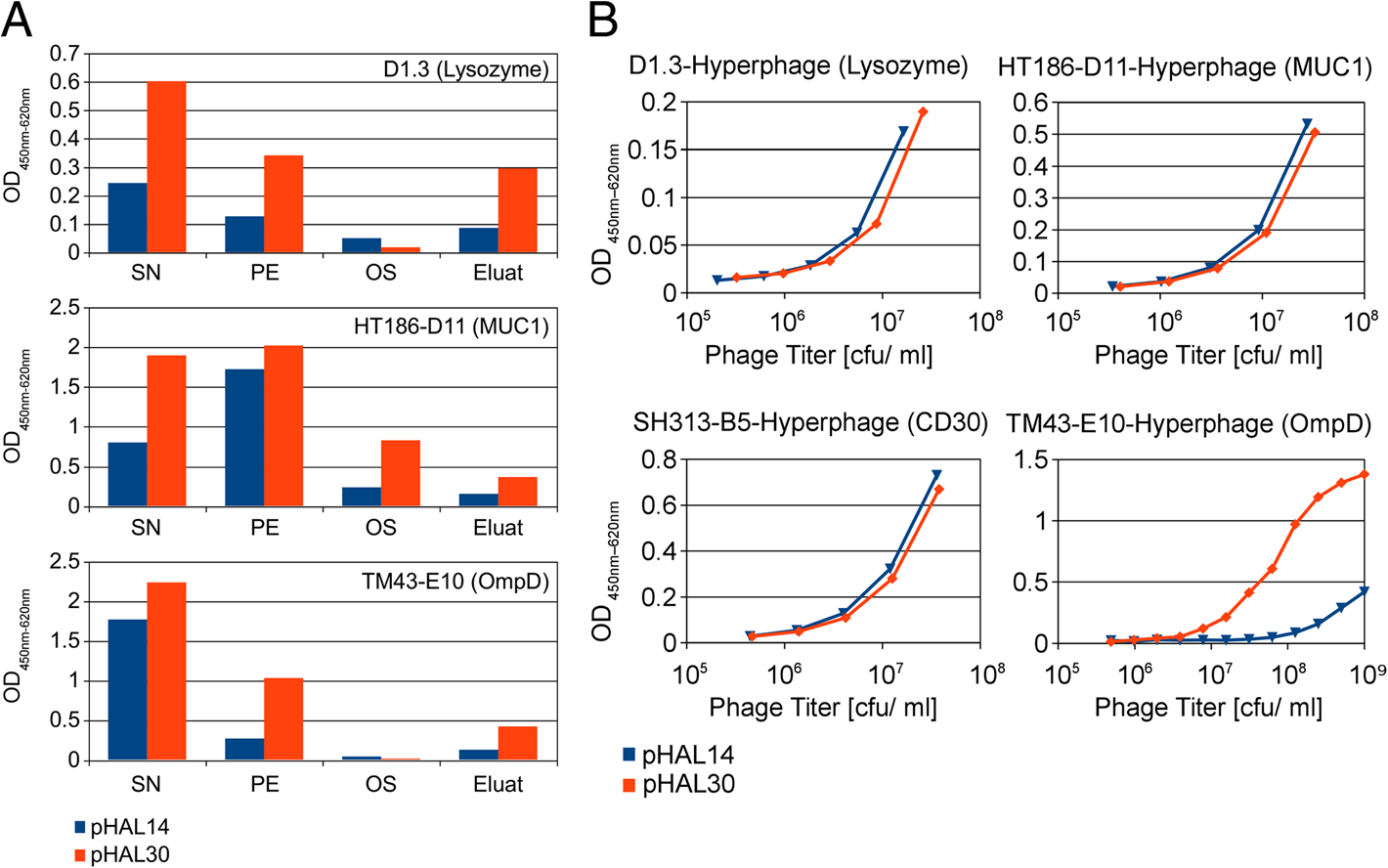
**Comparision of pHAL14 and pHAL30.** **(A)** Comparision of the production of soluble antibodies. SN supernatant, PE periplasmatic extract, OS osmotic shock fraction, Eluate after IMAC purification, OD_450nm-620nm_ optical density at 450 nm (620 nm reference), scFv antibody fragments analyzed: D1.3, HT186-D11 and TM43-E10, antigens: lysozyme, Muc-1 and OmpD. **(B)** Comparision of scFv-phage antigen binding. OD_450nm-620nm_ optical density at 450 nm (620 nm reference), scFv antibody fragments D1.3, HT186-D11, SH313-B5 and TM43-E10. antigens: lysozyme, MUC1, CD30 and OmpD.

A prerequisite for a good up to date antibody gene library is a phagemid that allows both, a high display level of functional antibody fragments on phage and a good expression of soluble antibodies for screening. The new design of the phagemid generation pHAL30 (Figure 1) is based on the pHAL14 [31]. In the first optimization step and independent of kappa or lambda light chains, the tag order in the pHAL30 phagemid was changed from the initial His/Myc-tag in pHAL14 to Myc/His tag. For evaluation of the expression capacity of both phagemids, three different soluble scFv antibody fragments were produced in 150 mL *E.coli* XL-1 Blue MRF’ culture for 4 h at 25°C and 250 rpm. Functional soluble scFv antibody fragments were quantified by antigen ELISA in the production supernatant, the periplasmatic extract, the osmotic shock fraction and the eluate after IMAC purification (Figure 2A). Except for the osmotic shock preparation, the production level with the pHAL30 phagemid was always significantly improved. After IMAC purification of the supernatants, the yields of HT186-D11 were 1 mg/L for pHAL14 and 2 mg/L for pHAL30, in case of TM43-E10 the yields were 1 mg/L for both vectors and for D1.3 the yields increased from 0.6 mg/L for pHAL14 to 1 mg/L for pHAL30. The production yields in the periplasmic fraction of scFvs HT186-D11 and TM43-E10 after IMAC purification were improved from 1.6 mg/L, and 0.9 mg/L with pHAL14 to 3.9 mg/L and 2.4 mg/L, respectively, with pHAL30.

Next, four different pHAL14 and pHAL30 phagemids were packaged with Hyperphage. The resulting phage titers were comparable among each other. They ranged from 6 × 10^9^ to 6 × 10^10^ cfu/ mL culture medium. The scFv display level on phage was identical, as confirmed by Western immunoblot (data not shown). Approximately 90% of g3p was fused to the scFv antibody fragments.

A functional comparison of pHAL14 and pHAL30 scFv-phage regarding antigen binding was performed by titration antigen ELISA (Figure 2B). The titration curves were similar for three different scFv-phage, further illustrating similar display level. For TM43-E10 more functional scFv were displayed on phage. Taken together, both phagemids are almost identical in phage display level and phage binding assays, but the pHAL30 phagemid provided a significant increase in production of soluble antibody fragments for screening which may improve the hit rate in initial screening assays.

### Influence of the phenylalanine residue in kappa CL on scFv phage display

Target independent, it was observed that scFv antibody fragments bearing a V_К_ light chain were less frequently isolated from HAL7/8 than those with a V_λ_ light chain [31,43]. A possible explanation could be a lower expression of antibody fragments with a V_К_ light chain. Therefore, the phagemid was further optimized by deleting the bulky C-terminal phenylalanine of the V_К_ light chain. First, the soluble production level of five known scFv fragments in pHAL14 and six known kappa scFv fragments in pHAL30 was analyzed and compared by Western immunoblot (data not shown). The deletion of the phenylalanine in the V_К_ light chain resulted in a higher production level for all tested scFv. Further expression analysis by Western immunblot included the comparison of 32 randomly chosen scFv fragments from the kappa HAL8 antibody gene library and their phenylalanine deletion version (Table 1). The expression levels were higher with the phenylalanine deletion mutants in 14 cases, slightly lower for three clones and in the same range for the remaining 15 clones.

### Construction of HAL9 and HAL10

Using blood samples obtained from 98 Caucasian, African, Indian and Chinese non-immunized human donors, the new antibody libraries HAL9 and HAL10 were constructed using the improved phagemid backbone pHAL30. In detail, blood lymphocytes from 54 Caucasian, African, Indian and Chinese non-immunized human donors were isolated for library construction. Lymphocyte mRNA was reverse transcribed to cDNA and the antibody gene repertoire was amplified in two steps. In the first step, VH and the full length light chains were amplified using a set of framework 1 forward primers and IgM, kappa and lambda constant domain reverse primers [40]. In the second step, specific restriction sites were incorporated for cloning into the pHAL30 phagemid vector. For the second amplification step of VH and lambda subfamilies the DNA antibody repertoire (1^st^ PCR material) from 44 donors of the previously constructed HAL7 was added for construction of the new HAL9 and HAL10 libraries. Both libraries contain the same VH repertoire (from 98 donors) but differ in their light chain repertoire. HAL9 includes all lambda subfamilies (from 98 donors), HAL10 all kappa subfamilies (from 54 donors) except the pseudogene encoding Vκ7. Cloning of the antibody genes was done in two steps. First, light chain genes were cloned with 1 × 10^9^ independent clones for each library. Then heavy chain genes were cloned in the light chain containing phagemid leading to a final repertoire size of the HAL9 and HAL10 scFv libraries of 1.04 × 10^10^ and 4.45 × 10^9^, respectively.

### Analysis of V-gene subfamily distribution

**Figure 3:**
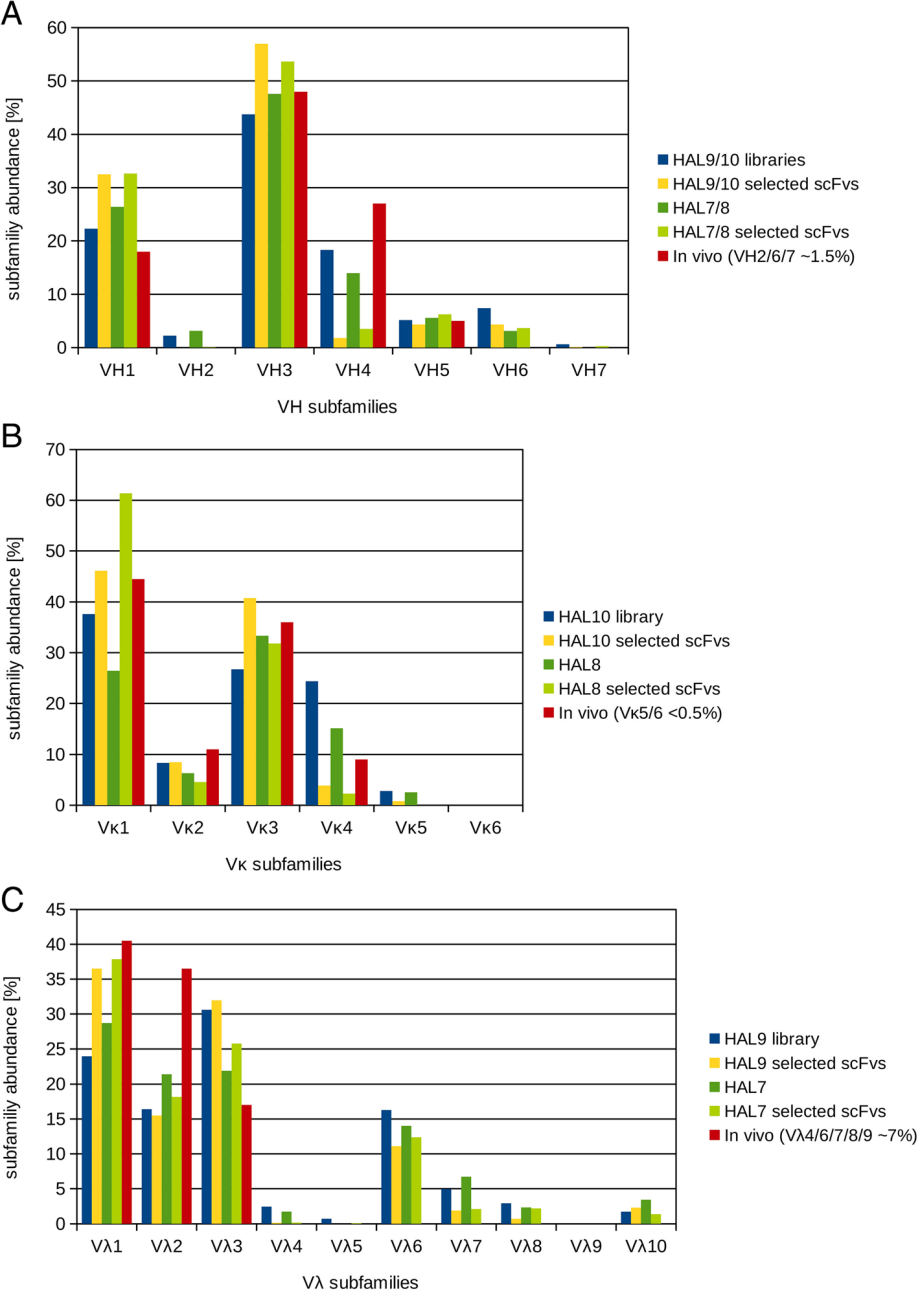
**Antibody subfamily distribution found in the HAL9/10 libraries, in the selected scFvs from the HAL9/10 libraries, in the HAL7/8 libraries [** 31 **] and** ***in vivo*** **[** 45 **]. (A)** Abundance of VH. **(B)** Abundance of Vκ. **(C)** Abundance of Vλ.

To assess the diversity of the libraries, bacterial colonies from library transformation plates were analyzed by colony PCR and sequencing. In total 827 full length scFv encoding sequences for HAL9 and 466 sequences for HAL10 were aligned to human germline V gene segments using VBASE2 [44]. To compare the initial diversity of the libraries with the germline usage after selection, 834 antibodies selected against 121 targets were analyzed. Of these selected antibodies from libraries HAL9 and HAL10, 15.6% were bearing a Vκ light chain compared to 4% of antibodies selected from HAL7/8. The antibody subfamily distribution of the unselected HAL7/8 libraries (367 sequences for HAL7 and 159 sequences for HAL8), the subfamily frequency of selected scFvs from HAL7/8 (1201 antibody sequences) and the *in vivo* distribution [45] are shown in Figure 3A-C. The heavy chain diversity of the unselected HAL9/10 libraries is similar to the subfamily distribution found *in vivo* (Figure 3A). VH1 and VH3 are the dominating VH subfamilies in the initial library as well as for the selected scFvs. VH4 shows a slight underrepresentation in the initial library, whereas the VH6 subfamily is overrepresented in all unselected and selected HAL libraries. Vκ1 and Vκ3 are the most abundant subfamilies, which is consistent with findings *in vivo* (Figure 3A). Vκ4 is overrepresented in the unselected HAL10 library but could not be retrieved after selection and in contrast Vκ1 was significantly enriched during selection. In the Vλ repertoire of HAL9, the Vλ1-3 are the most abundant subfamilies similar to the subfamily distribution found *in vivo* (Figure 3C), but the frequency of Vλ1-3 is lower compared to the *in vivo* frequency due to an overrepresentation of subfamilies Vλ4/6/7/8/10 which are found only in low frequency *in vivo*.

**Figure 4:**
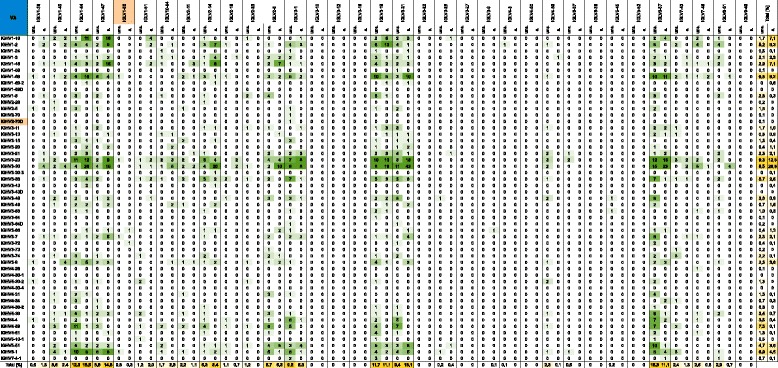
**Two-dimensional heatmap illustrating VH–Vλ gene pairing frequencies in the unselected HAL9 library (uns.) and in the selected scFvs from the HAL9 library (s.).** Frequency of all functional VH genes [46] and Vλ genes [47] are shown. Genes that are not confirmed to be functional, but were found in the library/were selected are marked in orange. 827 antibodies of the initial library and 704 selected scFvs were analyzed. The percentual amounts of each germline gene are given separetely for VH (rightmost columns) and Vλ (bottom row).

**Figure 5:**
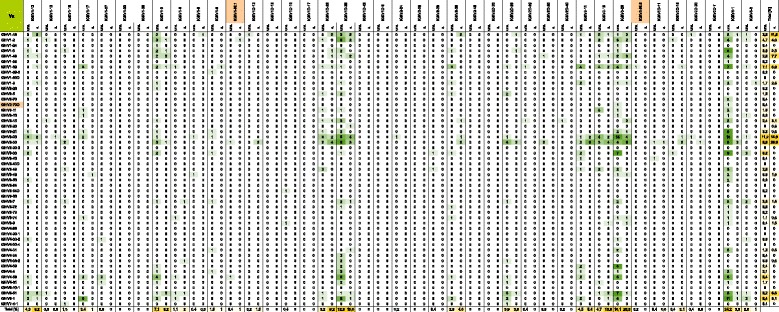
**Two-dimensional heatmap illustrating VH–Vκ gene pairing frequencies in the unselected HAL10 library (uns.) and in the selected scFvs from the HAL10 library (s.).** Frequency of all functional VH genes [46] and Vκ genes [48] are shown. Genes that are not confirmed to be functional, but were found in the library/were selected are marked in orange. Vκ6 genes are all classified as non-functional and not inclued in this overview. 466 antibodies of the initial library and 130 selected scFvs were analyzed. The percentual amounts of each germline gene are given separetely for VH (rightmost columns) and Vκ (bottom row).

Sequence analysis indicated a high antibody germline gene diversity for the heavy-chain and light-chain repertoires of the HAL9/10 libraries. The identified heavy chain repertoire in this limited sample already covered 46 of 50 functional VH genes (Figures 4 and 5). In addition, one VH gene (IGHV2-70D) occurred in the unselected libraries that has not yet been confirmed to be functional [46]. VH germline genes were observed in frequencies between 0.1% and 11.2% in the unselected HAL9 and HAL10 with no significant differences between the libraries. The comparison of the gene usage before and after selection shows a clear enrichment of some VH genes. The most abundant VH genes after selection were IGHV3-30, increased from 8.5% in the unselected libraries to 26.8% after selection, IGHV1-46, increased from 2.7% to 7.1% and IGHV1-18, increased from 2.1% to 7.8% after selection. The frequency of the remaining VH germline genes remained constant or was lowered after selection. Most strikingly, the usage of all VH4 genes was considerably reduced in selected antibodies. Only 6 of 9 VH4 germline genes were found again after selection with a ten-fold reduction in total frequency, decreasing from 18.3% in the unselected libraries to 1.8% after selection (Figure 3A and Figures 4 and 5).

24 of the 33 functional Vλ genes were found in the HAL9 library sample (Figure 4). One Vλ gene (IGLV1-50) was found in the unselected HAL9 as well in selected scFvs that is predicted to be not functional [47]. As seen for the heavy chain repertoire, the frequency of several Vλ germline genes changed during the selection process. Usage of the Vλ1 subfamily was increased after selection mostly due to a higher abundance of the IGLV1-47 germline gene, increasing from 5.8% in the unselected library to 14.5% after selection and a higher abundance of IGLV1-44, increasing from 12.3% to 15.5% (Figure 3C and Figure 4). In the Vλ2 subfamily, the frequency of the IGLV2-14 germline gene showed a slight increase from 5.3% to 8.4% after selection but the overall abundance of Vλ2 genes decreased due to a lower usage of the remaining germline genes. Three of nine different Vλ3 germline genes were retrieved in high frequency before and after selection: IGLV3-1, IGLV3-19 and IGLV2-21. The findings reflect the *in vivo* subfamily distribution, where IGLV3-1 and IGLV2-21 account for ~80% of Vλ3 [45]. After selection the frequency of IGLV3-1 was reduced from 9.2% to 5.3% and increased for IGLV3-21 from 9.4% to 15.1%. Usage of IGLV3-19 remained constant. The abundance of the most prevalent Vλ gene IGLV6-57 in the unselected HAL9 was decreased from 16.3% to 11.1% after selection.

In the HAL10 library 29 of 38 functional Vκ germline genes were observed (Figure 5). Two Vκ genes (IGKV1-NL1 and IGKV3-NL5) found in the unselected library were described to be only potentially functional [48]. The most abundant Vκ germline gene before selection was IGKV4-1 with a frequency of ~25%. This is a more than twofold higher prevalence than found *in vivo* [45] and usage of IGKV4-1 after selection was reduced to ~4%. The frequency of Vκ1 subfamily genes was raised from 37% in the unselected library to 48% after selection (Figure 3B and Figure 5). Three genes IGKV1-12, IGKV1-D33 and IGKV1-D39 increased from 4.4 to 9.2%, 3.3 to 9.2% and from 12.9 to 13.8%, respectively.

### VH and VL germline gene pairings

The abundance of heavy- and light-chain pairings that were observed in the unselected libraries and within selected antibodies are illustrated in Figure 4 for VH-Vλ combinations and in Figure 5 for VH-Vκ combinations. In the unselected libraries no particular VH-VL combination is dominating (Figure 4 and 5). The pairings are evenly distributed depending on their total abundance. VH-Vλ pairings were observed in frequencies between 0.1% and 1.8% in the unselected HAL9 with VH3-30/Vλ6-57 being the most abundant combination. In the unselected HAL10 pairing frequencies up to 2.8% were observed for VH-Vκ due to the high abundance of the IGKV4-1 germline gene. However, after 121 individual selections a clear preference for particular VH-VL pairings was detected. For VH-Vλ pairings the most prevalent combination was VH3-30/Vλ3-21 which was observed in 6.1% of the selected antibodies. The λ3-21 gene showed a preference for pairing with VH3 germline genes. The VH3-30 germline gene was by far the most abundant VH gene after selection and paired with nearly all light chain genes in high frequency. Interestingly, the light chain gene Vλ8-61 paired almost exclusively with VH3-30. The remaining Vλ germline genes showed preferential pairing with the VH1 and VH3 germline genes and with lower frequency also to the VH6-1 germline gene. A second VH3 subfamily gene, VH3-23, was also observed in high frequency after selection. Similar to VH3-30 no clear preference in pairing with any Vλ genes segment was seen. For three VH1 genes a more distinct preference for single Vλ genes was observed. The combinations VH1-46/Vλ1-47 and VH1-69/Vλ1-44 were enriched to 2.3% and the combination VH1-18/Vλ1-47 was enriched to 2.6%. The five most common VH-Vλ combinations (VH3-30/Vλ3-21, VH3-30/Vλ1-44, VH3-30/Vλ6-57, VH3-30/Vλ2-14 and VH3-30/Vλ3-14) account for nearly 20% of all selected antibodies.

An enrichment of the VH3-30 germline gene after selection was also observed for the VH-Vκ gene pairing. Furthermore a high frequency of Vκ1 and Vκ2 genes occurred after selection, but due to the relatively small sample size of selected antibodies bearing a kappa light chain no significant conclusion can be made about enrichment of single VH-Vκ combinations.

### CDR3 length and amino acid distribution of light-chain and heavy-chain

**Figure 6:**
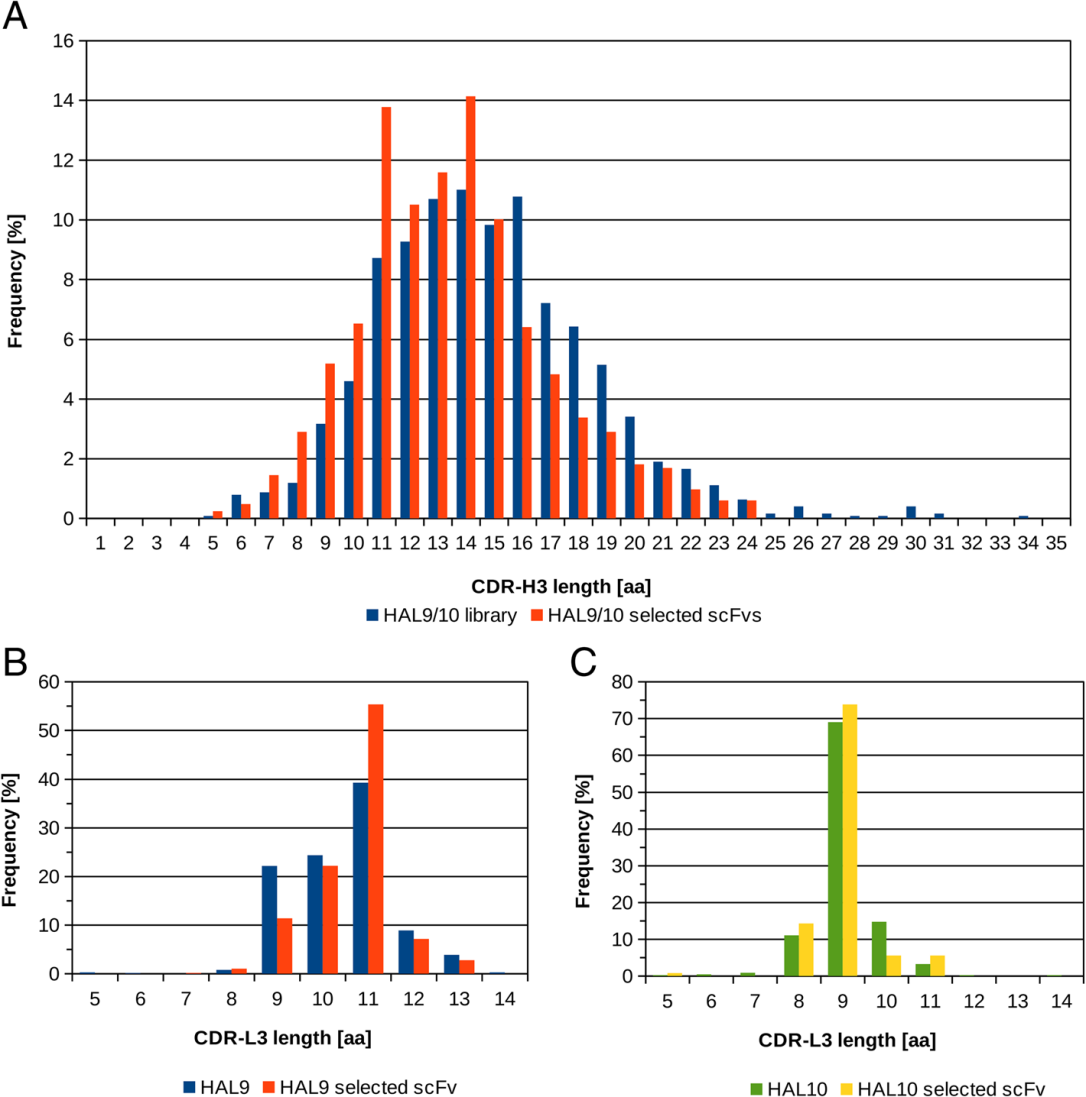
**Heavy and light chain CDR length distribution. (A)** CDR-H3 length distribution in HAL9/10. CDR-H3 lengths in unselected library (blue bars, analysis of 1262 sequences) and in selected scFvs from HAL9/10 (red bars, analysis of 828 sequences). **(B)** CDR-L3 length distribution in HAL9. CDR-L3 lengths in unselected library (blue bars, analysis of 776 sequences) and in selected scFvs from HAL9 (red bars, analysis of 685 sequences). **(C)** CDR-L3 length distribution in HAL10. CDR-L3 lengths in unselected library (green bars, analysis of 461 sequences) and in selected scFvs from HAL9 (yellow bars, analysis of 126 sequences).

Length and amino acid distribution of CDR3-L and CDR3-H of the HAL9 and HAL10 libraries were analyzed. For example, 1262 CDR3-H sequences of the unselected libraries and 828 sequences of selected antibodies were analyzed and compared. The CDR3-H length distribution before and after selection is depicted in Figure 6A. In the unselected libraries, the CDR3-H length ranges from 5 to 35 aa with a median length of about 14 aa. CDR3-H longer than 24 aa are occurring only in low frequency. After selection, the distribution is very slightly shifted towards a CDR3-H length with a median of 13 aa. CDR3 length of 11 – 15 aa are found in high frequency after selection but CDR3-H length longer than 24 aa were not detected. Long CDR-H3 sequences are often internally stabilized by disulfide bridges in a very characteristic manner. A first internal cysteine is always followed by four amino acids - often serine and glycine rich - and completed by the second cysteine (data not shown).

**Figure 7:**
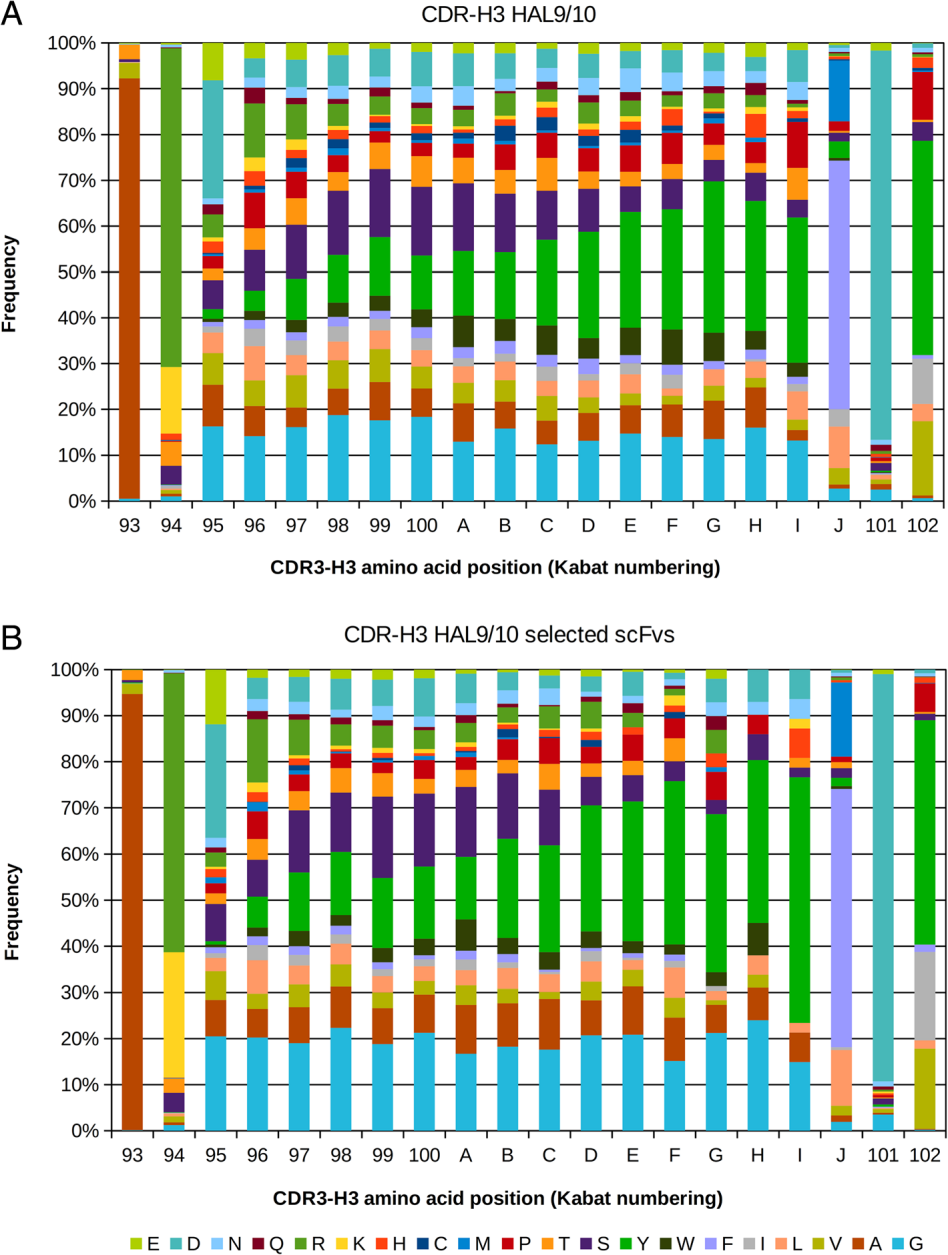
**Heavy chain CDR amino acid distribution. (A)** CDR-H3 amino acid distribution in unselected HAL9/10 (analysis of 1262 sequences). **(B)** CDR-H3 amino acid distribution in selected scFvs from HAL9/10 (analysis of 828 sequences). Distribution of amino acids at each position of CDR-H3 in Kabat numbering. Amino acids 100-J, 101 and 103 correspond to the last 3 residues of each CDR3-H3.

The CDR3-H amino acid composition was analyzed for the unselected HAL9/10 libraries (Figure 7A). The amino acid usage at each position is shown for CDRs with up to 20 aa in length. The CDR3-H often starts with an alanine and arginine in the first two positions and throughout the whole CDR the amino acids glutamate, serine, tyrosine and glycine are favored. At the last three positions the “FDY” motive of the IGHJ4 segment is often found. Significantly, the CDR3-H amino acid usage found in selected scFvs is largely the same compared to the amino acid usage in the initial libraries (Figure 7B).

For the CDR3-L length distribution of HAL9 (lambda light chain) before and after selection 776 sequences of the unselected library and 685 sequences of selected antibodies were analyzed (Figure 6B). In the unselected library the CDR3 length ranges from 8 to 14 aa with a median length of about 11 aa. The most common CDR3 length for lambda light chains are 9, 10 or 11 aa that account together for more than 80% of all found CDR3-L lengths. After selection, CDRs with a length of 11 aa were found more frequently and the occurrence of CDRs with a length of 9 aa was decreased. CDR3-L length distribution of HAL10 (kappa light chain) was analyzed with 461 sequences of the unselected library and 126 sequences of selected antibodies (Figure 6C). In the unselected library the CDR3 length ranges from 7 to 11 aa. Most kappa light chains are bearing a CDR3 with a length of 9 aa (~70%) and the length distribution did not change significantly after selection.

**Figure 8:**
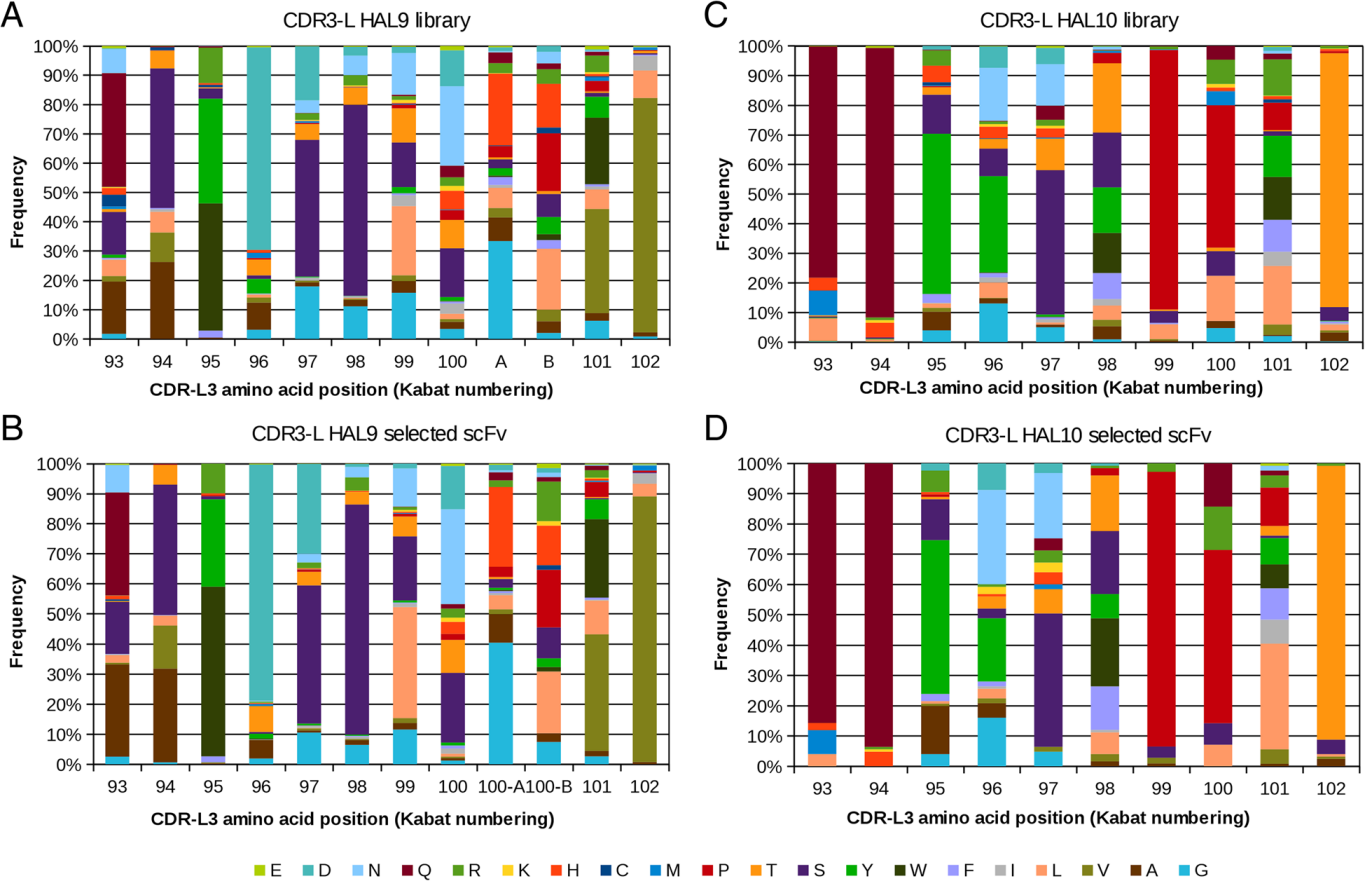
**Light chain CDR amino acid distribution. (A)** CDR-L3 amino acid distribution in HAL9 (analysis of 776 sequences). **(B)** CDR-L3 amino acid distribution in selected scFvs from HAL9 (analysis of 685 sequences). **(C)** CDR-L3 amino acid distribution in HAL10 (analysis of 461 sequences). **(D)** CDR-L3 amino acid distribution in selected scFvs from HAL10 (analysis of 126 sequences). Distribution of amino acids at each position of CDR-L3 in Kabat numbering.

The CDR3-L amino acid distribution of the unselected libraries was analyzed separately for lambda and kappa light chains. The amino acid usage at each position is shown for CDRs with up to 12 aa in length for lambda light chains (Figure 8A) and for CDRs with up to 10 aa in length for kappa light chains (Figure 8C). At most positions of the light chain CDR3, the usage of amino acids is restricted, e.g. 70% of antibodies have a aspartate at lambda CDR3 position 96, or 90% have a proline at kappa CDR3 position 99, but there is still a quite high diversity found in amino acid usage throughout the whole CDR. Again, the CDR3-L amino acid distribution found in selected scFvs is almost the same when compared to the amino acid usage of the initial libraries HAL9/10 (Figure 8B, D).

## Discussion

Since the development of phagemids for antibody phage display by Breitling et al. [49], the technology was continuously further developed.

The most used phage display vectors have the tag order His-Myc when using both tags, e.g. pCANTAB3his [50], pCES [51], pHEN2 [52], pHAL14 [31], pIT2 [53], pMod1 [54] or pMID21 [20]. To our knowledge, there is only one phage display vector using the opposite orientation: pSD3 [55]. Comparing both tag orders, the Myc-His order (pHAL30) results in improved production of functional scFv compared to His-Myc (pHAL14) whereas the display of functional scFv on phage is not effected.

Using a kappa (HAL4 or HAL8) and a lambda (HAL7) antibody gene library in the same selection, we have observed that the output of selected kappa antibodies was always lower compared to lambda antibodies [31,43]. Løset et al. and Tiller et al. also describe a lower production yield of kappa scFvs or Fabs compared to lambda antibodies that confirms our observation with the HAL libraries [45,56]. The scFv design in pHAL14 included the first six amino acids of CH1 as part of the linker and the first 16 amino acids of CL lambda, respectively the first nine amino acids of CL kappa. The last amino acid in CL kappa used in the scFv design is a phenylalanine. Phenylalanine is encoded by only two codons which may lead to a reduced translation and has a bulky hydrophobic benzol side group which may hamper solubility. In projects with macaque immune libraries using also pHAL14 and derived pHAL vectors, the phenylalanine was omitted. The selected kappa scFv were produced well, leading to a generation of kappa scFv against different targets [8,57-62]. For human scFv, deletion of phenylalanine improved the expression rate of known kappa antibodies and randomly chosen kappa clones. This modification increased the rate of selected kappa antibodies from 4% using HAL4/7/8 to 15.6% when using HAL9/10.

Using the novel vector pHAL30 and scFv design, two new universal human naive antibody gene libraries were constructed. The theoretical diversity of the HAL9/10 libraries is with 1.5 × 10^10^ independent clones in the same range like other published scFv or Fab libraries with a theoretical diversity in the range of 1x10^10^-1x10^11^, e.g. the naive McCafferty scFv library (1.1x10^10^ independent clones) [63], the naive Pfizer scFv library (3.1 × 10^10^) [64], the naive CAT2.0 scFv library (1.29 × 10^11^) [65], the naive/synthetic Dyax Fab (3.5 × 10^10^) [20] library, the synthetic Ylanthia Fab library (1.3 × 10^11^) [45] or very recently published the PHILODiamond scFv library (4 × 10^10^) [66].

The representation of antibody subfamilies in the HAL9/10 libraries is comparable with the HAL7/8 library [31,43], it differs only in some minor points, e.g. VH4, Vκ4 and Vλ4 are more abundant in HAL9/10. In general, VH gene usage is dominated by VH3 and VH1. Dominant VL are usually Vκ1, Vκ3, Vκ4 and Vκ2 and Vλ by Vλ3, Vλ1, Vλ2 and Vλ6. In the new libraries, the distribution of V genes is comparable with their frequency *in vivo* [45] but with following exceptions. The subfamily VH4 is more abundant *in vivo* whereas Vκ4 and Vλ6 are more frequent in HAL antibody gene libraries and rare *in vivo*. The main difference is the frequency of Vλ1 > Vλ2 > Vλ3 *in vivo* compared to Vλ3 > Vλ1 > Vλ2 in HAL9/10. The Ylanthia library [45] fits perfectly with the *in vivo* distribution. The distribution of V genes for VH and Vκ of HAL9/10 is in accordance with the Pfizer library [64]. A comparison of Vλ with HAL9 is not possible, because the Pfizer library lacks most Vλ genes. Compared to HAL9/10, the distribution of V genes in the CAT2.0 library [65] differs more from *in vivo* situation. Here, VH is dominated by VH1 and Vκ is dominated by one Vκ1 gene. With ~14 amino acids, the average HAL9/10 CDR-H3 length is longer than in the naive Pfizer [64] or synthetic Ylanthia [45] libraries (~11 amino acids), but slightly shorter compared to the Dyax library with ~15 amino acids [20]. In addition more very long CDR-H3 (>20 amino acids) are found in HAL9/10 compared to the Pfizer library. The average length of CDR-L3 for λ is eleven amino acids and therefore two amino acids longer as the average length of CDR-L3 for κ. In HAL9/10, 19 or 20 different aa are occurring at each CDR-H3 position whereas for Ylanthia 13 to 17 different aa are possible at each position, planned by rational design [45]. In Ylanthia, the CDR-H3 is dominated by serine, tyrosine and glycine (~40% frequency). In the naive HAL9/10 libraries, the amino acid repertoire in CDR-H3 is more balanced, but the frequency of tyrosine increases from N’terminus to C’terminus as well (aa 95 - 100i).

Significantly, within the group of selected scFvs, the representation of VH antibody subfamilies is largely in accordance with the McCafferty library [63] and Pfizer library [64], but not with the CAT2.0 library [65], where VH is dominated by VH1 > VH3 compared to VH3 > VH1 for McCafferty, HAL9/10 or Pfizer library. For Vκ, more Vκ1 than Vκ3 were selected from HAL10 or McCafferty, whereas from Pfizer or CAT2.0 mostly Vκ3 were selected. Regarding Vλ, here Vλ1-3 and 6 are mainly isolated from McCaffery and HAL9, whereas Vλ6 is rarely selected from CAT2.0. The frequency of selected VH4, Vκ4 and Vλ7 antibodies from HAL9/10 is very low compared with the frequency in the initial library. The same was observed for HAL7/8 [31] and CAT2.0 [65]. The VH4-34 antibody gene is described as toxic for B-cells [67]. As consequence, two VH4-34 and VH4-59 genes were excluded when constructing the synthetic Ylanthia library, despite the fact, that these VH4 genes are very frequent in the natural human antibody repertoire [45]. Remarkably, a majority of antibodies selected from macaque immune antibody gene libraries against botulinum A toxin are VH4 antibodies, especially VH4-59 [59]. The macaque antibody genes are very similar to their human counterparts. Therefore, the VH4 gene family may be preadapted to some targets, or at least best suited to structurally complement the respective epitopes.

The average CDR length of CDR-H3 is only very slightly shorter after selection compared with the initial library, presumably due to a better growth of bacteria producing shorter antibody genes during antibody selection. The length of the CDR-L3 does not significantly differ in selected antibodies. The CDR composition in the CDR-L3 is completely different between λ and κ light chains, giving a hint why two different kinds of light chains have been evolved. Very importantly, the frequency of amino acids at each CDR3 position does not significantly change after selection, clearly demonstrating that any bacterial expression bias is minor, and all amino acids used in the naive human gene repertoire can be found in selected, functional antibodies.

Some VH-genes are preferred after selection, e.g. VH1-18, VH1-46, VH3-23, VH3-30. These are also V-genes often used in Ylanthia [45] and CAT2.0 [65]. Notably, the very common VH3-23 gene results in a low IgG1 expression rate, but is most frequent in the natural human antibody repertoire [45]. This V-gene is a very stable and was used also for semisynthetic libraries, e.g. Tomlinson Library [53]. For κ light chain, IGKV1-12, IGKV1D-33, IGKV1D-39 and IGKV3-20 are selected frequently. IGKV3-20 is the most used κ gene in the natural repertoire and is used for Ylanthia [45]. The λ V-genes IGLV1-44, IGLV1-47, IGLV2-14, IGLV3-19, IGLV3-21 and IGLV6-57 were most frequently found after HAL selection. IGLV2-14 is the most used λ V-gene in the natural antibody repertoire and is used for Ylanthia, IGLV1-44 and IGLV1-47 are also often used in nature, but IGLV1-44 was excluded from the Ylanthia design [45].

## Conclusion

The naive antibody gene libraries HAL9/10 have unique features compared to the libraries published earlier. The libraries have a slightly greater maximum diversity than the McCafferty library, they comprise more lambda V-genes and have longer CDR-H3 compared to the Pfizer library, the distribution of V-genes resembles more the natural antibody repertoire compared to CAT2.0 and they show more CDR-H3 diversity than Ylanthia. The successful generation of >800 antibodies against >100 targets with overwhelming success and in short time with miniaturized systems illustrates the huge advances in human library construction and use achieved since the first description of this method.
